# A Survey of the Microbiome, Culturome and ARG Profile of a Cohort of Chronic Diabetic Foot Lesions

**DOI:** 10.1111/apm.70203

**Published:** 2026-04-16

**Authors:** Karolina I. Pyrzanowska, Elspeth N. Smith, Chandra Ramalingam, Marni Greig, Rajiv A. Gandhi, Susan Heyes, Debora Sanger, Tracy Smith, David G. Partridge, Graham P. Stafford

**Affiliations:** ^1^ School of Clinical Dentistry University of Sheffield Sheffield UK; ^2^ Department of Diabetes and Endocrinology Sheffield Teaching Hospitals NHS Foundation Trust, Northern General Hospital Sheffield UK; ^3^ Florey Institute of Infection Sheffield UK; ^4^ Department of Laboratory Medicine, Sheffield Teaching Hospitals NHS Foundation Trust, Northern General Hospital Sheffield UK

**Keywords:** diabetic foot ulcers, microbiome, Oxford nanopore sequencing, polymicrobial infections

## Abstract

This study aimed to analyse the microbiome of chronic infected diabetic foot ulcers (DFUs) using parallel methods: traditional culture (the culturome), 16S rRNA gene sequencing (the microbiome) as well as the Antibiotic Resistance Gene (ARG) profile of isolated strains. Swab samples were collected in parallel from affected ulcers. The microbiome sequencing results identified that all patients had a polymicrobial flora with the five most frequent genus level OTUs as *Escherichia*, *Staphylococcus*, *Streptococcus*, *Pseudomonas* and the anaerobe *Anaerococcus*. Microbiological culture from the same swabs identified multiple species in all but two patient samples and revealed the most common isolates as CoNS *Staphylococcus* (17%), *Enterococcus faecalis* (14.3%), *Corynebacterium* spp. (10.7%), *Anaerococcus* spp. (7%), *Staphylococcus aureus* (8%) and *Pseudomonas aeruginosa* (6%). Enteric pathogens such as *Klebsiella* spp. were also frequently isolated. Attempts to revive anaerobes were largely unsuccessful, identifying a limitation in clinical microbiology storage protocols. Genome sequencing of 55 isolates revealed a high number of ARGs relating to β‐lactams and tetracyclines, indicating multi‐drug‐resistant organisms (MDROs). This was confirmed by phenotypic antimicrobial susceptibility data that included several highly resistant Gram‐negative bacteria. Overall, our data add to the picture of DFU microbiome as complex and displaying high levels of anti‐microbial resistance (AMR).

## 1 Introduction

Diabetes mellitus affects an estimated 4.9 million people in the United Kingdom and an estimated 537 million worldwide [1, 2]. Approximately 25% of diabetics will also develop a diabetic foot ulcer (DFU) in their life‐time. Once infected, 20% of DFUs with moderate to severe infections result in amputations, with evidence of poor 5‐year survival worse than most cancers in the United Kingdom (i.e., as high as 70% mortality), [3, 4, 5, 6, 7]. In the United Kingdom, complications related to diabetes, particularly those involving foot problems, incur an annual cost of £1 billion [3]. This figure includes both direct medical costs and expenses linked to disability and extended hospital stays. Overall, this expenditure represents around 1% of the total NHS budget and surpasses the combined expenditure on prostate, breast and lung cancer. Notably, at least 50% of DFU are infected at first clinical presentation. As a result, DFU patients receive multiple, protracted courses of antimicrobial treatment, which in turn promotes colonisation and/or infection with multidrug‐resistant organisms (MDROs) [8, 9].

The causative organisms of diabetic foot ulcer infections are historically considered to be *Staphylococcus*, *Streptococcus*, *Proteobacteria* and *Pseudomonas* species [10, 11, 12]. Among these, *Staphylococcus* species are the among the most frequently isolated in infected foot ulcers [13, 14, 15]. However, the frequency of ‘resistant’ or problematic infections and the recent availability of non‐culture‐based approaches suggests that most infections are polymicrobial and monomicrobial infections rarer [13, 16, 17, 18]. Organisms previously considered commensals are now associated with worse outcomes [19] and frequently complicate management due to multiply resistant antimicrobial profiles, including CoNS, Enterococci, Corynebacteria and Streptococci [18, 20, 21]. It has previously been established that chronic, harder to treat DFUs, including those leading to surgical intervention, include higher levels of anaerobic bacteria, for example *Finegoldia* spp., Anaerococci and Bacterioidia [22]. However, these and other anaerobes in general are hard to culture and preserve leading to a historical under‐estimation of their prevalence [23, 24, 25]. The polymicrobial and diverse microflora of DFU, makes appropriate antibiotic prescribing challenging, especially due to the rise in antibiotic resistant bacteria that are common in DFU [26, 27]. Additionally, antimicrobial resistance (AMR) presents a significant global public health challenge, rendering current therapies ineffective and posing life‐threatening risks to those afflicted by infections from multidrug‐resistant organisms [8, 9].

All of this evidence suggests that a fuller understanding of the complexity within the wound microbiome is essential [17]. Hence, this study set out to improve our understanding of the DFU microbiome by analysing swabs from infected sites of chronic heel ulcers to assess the microbiome associated with the diseased state by sequencing and, in parallel, standard clinical laboratory culture methods. Isolates were genome sequenced and their antibiotic resistance genes (ARGs) and AMR phenotypes investigated and compared.

## 2 Methods

### 2.1 Patient and Sample Collection Information

DFU heel ulcers were swabbed by trained clinical personnel under NHS ethics obtained by Dr. David Partridge (REC reference: [21]/SC/0338 and IRAS project ID: 299735). Patients over 18 years with Grade 3–4 DFU according to IDSA/IWG criteria [28] and willing to consent were selected. All attended the Sheffield Northern General Diabetes multidisciplinary foot clinic. Of the 30 patients swabbed, 19/30 were taking antibiotics for unresolved infections, with median ulcer durations of 8 weeks. Deep ulcer swabs were taken after saline cleansing of lesions to remove superficial slough. Patients were swabbed twice (in the same place) with standard cotton swabs in charcoal transport medium (for clinical standard culture, see below) or using Σ–MM swabs (for DNA extraction). To remove the possibility that the order of swabbing might influence results, we alternated the order of collection of the culture and DNA swabs. Σ–MM swabs were placed in their proprietary DNA preservation solution and stored at −80°C before processing for DNA extraction. Swabs for microbiological analysis were processed in the microbiology laboratory medicine department in line with the UK‐National Standards for Microbiological Investigation (UK‐SMI B11 (typically 1 x Standard blood agar‐ 48 h Co2, 1 x MacConkey 24 h air, 1 x SAB‐DEX 48 h air (derogation from SMI); 1 x Fastidious Anaerobe blood agar 48 h and 5d)). All cultured isolates were speciated by MALDI‐TOF MS (MALDI Biotyper sirius System) in line with UK Standards for Microbiological Investigation. Antibiotic susceptibility was tested by routine laboratory methods at the UKAS accredited Microbiology department, Directorate of Laboratory Medicine, Northern General Hospital, according to EUCAST breakpoints. Isolates were then stored at −80°C in media plus 15% glycerol (100 μL on beads). Alongside swabs, patient information regarding antibiotic treatment and the details of this treatment were collected. Culture data were also presented as a heatmap (heatmap2‐R‐studio (Average Linkage method)).

### 2.2 DNA Extraction, Genome Sequencing and Analysis

Strains were streaked to single colonies onto Brain Heart Infusion agar (BHI) (NeoGen) or Fastidious anaerobe agar (NeoGen) supplemented with 5% horse blood and grown overnight in a 37°C aerobic incubator, 37°C CO_2_ incubator or in a Don‐Whitley MiniMACS anaerobic chamber (CO_2_, Nitrogen, Hydrogen) as appropriate. DNA extraction was performed using a Wizard DNA purification kit (Promega, USA), according to the manufacturer's instructions. DNA samples were quantified using the Qubit high‐sensitivity (HS) dsDNA assay kit (ThermoFisher, USA) and measured using a Qubit 2 Fluorometer (Invitrogen, USA). DNA was stored at −20°C.

Clinical strain genomic DNA was sent to MicrobesNG (Birmingham, UK) for short‐read Illumina sequencing and genome assembly. DNA was sequenced on an Illumina NovaSeq 6000 (Illumina, San Diego, USA) using a 250 bp paired end protocol. Trimmomatic (v0.30) was used to trim reads with a sliding window quality cutoff of Q15. *De novo* assembly was performed on samples using SPAdes (v3.7) and contigs annotated using Prokka (v1.11).

Further sequence analysis was carried out using tools on the Galaxy Europe web platform (v23.2). This included Multil‐locus sequence typing (PubMLST), RG identification using AMRFinderPlus [29] and StarAMR/Resfinder [30, 31].

The genomes of the clinical DFU strain collection were imported into the Kbase system (Version 2.7.11) to build a genome set and a phylogenetic tree constructed using Insert Genome Into SpeciesTree (Version 2.2.0) [32]. Bootstrap confidence values were generated using 1000 replicates. The tree was visualised and edited in Interactive Tree Of Life (ITOL, Version 7.1) [33].

### 2.3 16S DNA Amplicon Sequencing and Analysis

DNA was extracted from Σ–MM swabs using a QIAmp DNA mini kit (Qiagen, UK) with a control prepared using a plain Σ–MM swab. In brief, 500 μL of the preservation solution was transferred into a clean tube and 150 μL lysozyme (10 mg/mL), 6 μL mutanolysin (25,000 U/mL) and 3 μL lysostaphin were added and incubated at 37°C for 1 h. Subsequently, 40 μL proteinase K, 8 μL RNAse A (100 mg/mL) were added and incubated at 56°C for 10 mins. The manufacturers’ instructions were then followed. DNA was stored at 4°C and used for Oxford Nanopore 16S Barcoding Kit library preparation using the Barcoding Kit 1–24 (SQK‐16S024) with primers 27F (5′‐AGAGTTTGATCMTGGCTCAG‐3′) and 1492R (5′‐CGGTTACCTTGTTACGACTT‐3′).

The reactions for 16S polymerase chain reaction (PCR) comprised: 12.5 μL of LongAmp Taq 2X Master Mix (NEB, UK), 5ul of 16S Barcoding Kit 1–24 primer (SQK‐16S024), 5.5ul of nuclease‐free water, and 2ul of template gDNA (20 ng) to a final volume of 25ul. Alongside, PCR reactions for a negative no template control, DNA extracted from a negative control swab (blank Σ–MMTM extracted using the same protocol as regular swab) and a ZymoBiomics Microbial Community DNA Standard (Zymo Research, UK) were also performed. PCR was carried out with denaturation at 95°C (20s), annealing at 55°C (30s) and extension at 65°C (2mins) for 30 cycles. PCR products were analysed using ethidium bromide Agarose gel electrophoresis.

Following the PCR step, each reaction was transferred to a separate 1.5 mL Eppendorf DNA LoBind tube before and further processed using Agencourt AMPure XP beads. Each sample was quantified using a Qubit 2 Fluorometer (Qubit dsDNA HS Assay Kit (ThermoFisher, USA)). All barcoded libraries were pooled to a total of 50–100 fmoles in 10 μL of 10 mM Tris–HCl pH 8, 50 mM NaCl. 1 μL of rapid adapter (RAP) was added and incubated at room temperature for 5 mins. Priming and loading the flow cell was carried out using the Oxford Nanopore Technologies Flow Cell Priming Kit (EXP‐FLP002). MinION reads were basecalled (Guppy v.6.4.6) using a MinION MK1C (ONT v9.4.1 flow‐cells, Oxford Nanopore Technologies, UK) and FASTq reads extracted. Barcodes and adapters were trimmed and demultiplexed using Porechop (https://github.com/rrwick/Porechop/) on the Galaxy Europe server. The processed reads were then analysed using Mothur (Version 1.48.0; [34]), and the results were visualised with RStudio 2023.12.1 + 402 for MacOS (http://www.rstudio.com/).

All data are available via the European Nucleotide Archive (ENA) with project number PRJEB98719 for microbiome and available via NCBI with project number PRJNA1254668 for strain collection genomic data. All raw data are available in Tables S1 and S2.

## 3 Results and Discussion

### 3.1 Patient Characteristics and Demographics

Patients included in the study averaged 66 years old; a cohort similar to other studies in the United Kingdom, the United States and Europe [35, 36, 37, 38]. 77% of patients were male and of British White (Caucasian) ethnicity, reflecting the demographics of consenting patients attending the foot clinic. All patients swabbed had IDSA/IWG grade 3 or 4 infection, with 19 taking antibiotics for unresolved infections with median ulcer durations of 8 weeks. Complete metadata are presented in Table 1.

**TABLE 1:** DFU patient demographics: Age, ethnicity and sex distribution. The table presents the demographic information of a total patient cohort consisting of swabs from 27 unique patients and a total of 33 culture swabs as six patients were sampled twice.

Patient’ demographics	
Total patient cohort (*n* = 27)	
Age (mean, years)	65.61
Ethnicity	
White	100.00
Black	0.00
Asian	0.00
Mixed	0.00
Sex	
Female	22.22
Male	77.78

### 3.2 The 16S Microbiome of DFU Patient Cohort

Of the patient samples collected, 21 swabs from 19 patients were used for sequence analysis. However, nine samples were excluded due to low quantity or quality of DNA, or insufficient read numbers after sequencing, that is samples with fewer than 200 classified reads. We acknowledge that we have set our threshold relatively low and did not reach saturation in terms of diversity. However, we felt it important to maximise the data extracted from this valuable cohort. To ensure that even these low read counts represent real detection of sequence OTUs, we also conducted DNA extraction and ‘kit‐ome’ controls, where reads were essentially zero to ensure our analysis did not include contaminating reads and OTUs. We also acknowledge that the absence of PMA treatment of our samples means we potentially amplified live and non‐viable bacteria, but since our 16S have significant crossover with the culture data, we believe this did not skew the data.

**FIGURE 1:**
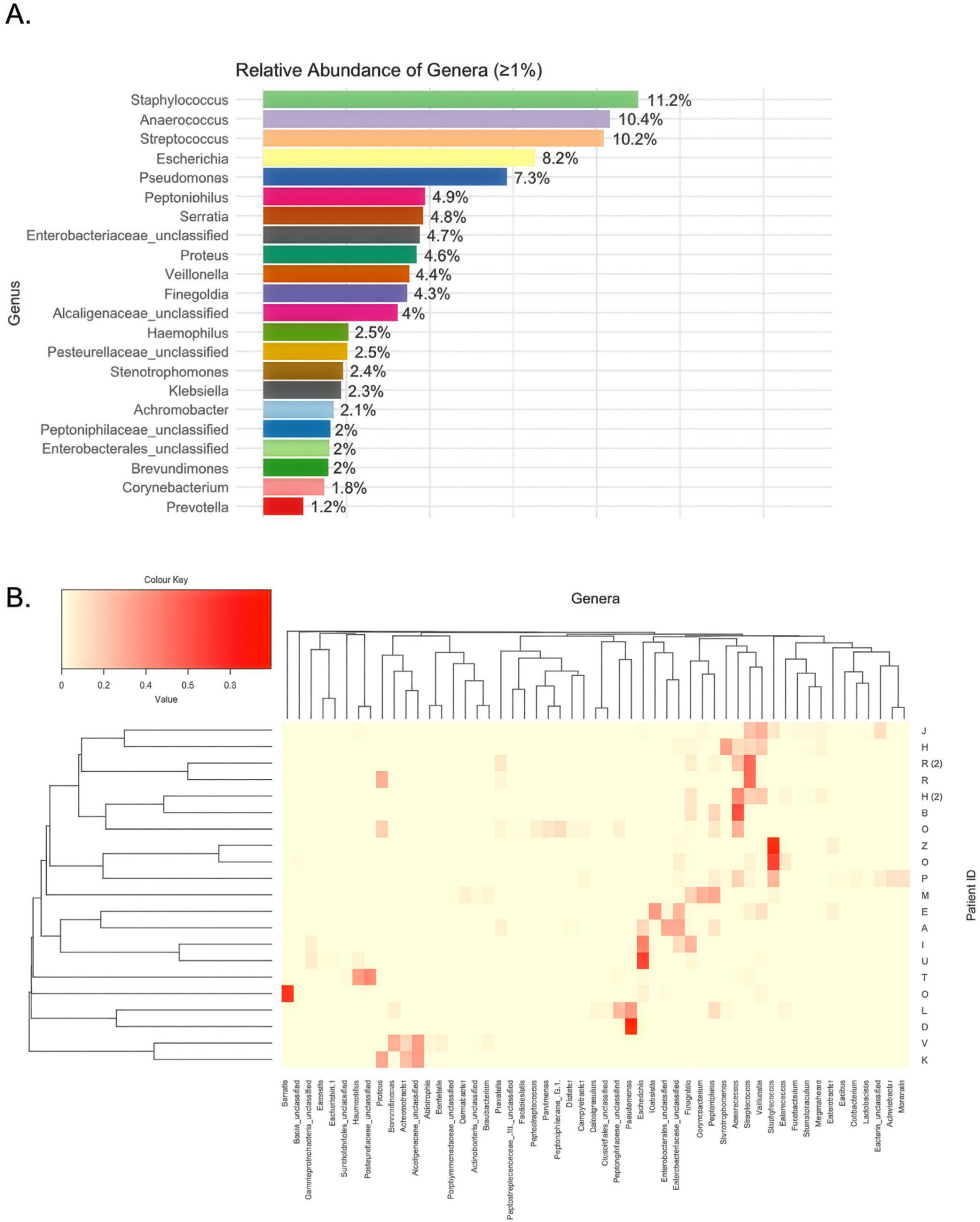
Summary data for 16S microbiome sequencing from chronic DFU swabs. (A) Represents the relative abundance of genera across all samples after filtering out those with a relative abundance below 1%. The percentages indicate the proportion of each genus relative to the total bacterial community, highlighting the dominant taxa in the dataset while minimizing the influence of low abundance genera. (B) Heatmap displaying the Nanopore sequencing‐based microbial composition of 21 samples from 19 patients. The data were hierarchically clustered based on Bray–Curtis dissimilarity at the genus level with operational taxonomic unit assignments derived from trimmed sequence data. Only genera with a maximum relative abundance of ≥ 1% were included. Patients H and R provided two swab samples each, represented as H (2) and R (2). Patient IDs are represented on the right. Clustering and visualisation were performed for both rows (patients) and columns (genera) in R Studio using the heatmap.2() function.

Following mothur analysis we classified to genus level (since utilised ONT v9.4.1 flow‐cells), with the most commonly attributed OTU genera outlined in Figure 1A (count data: Tables [Link], [Link]). A total of 120 genera were classified in total across the patient samples at an abundance > 1%. The most frequently identified genera (mean relative abundance) were *Staphylococcus* (11.2%), *Anaerococcus* (10.4%), *Streptococcus* (10.2%), *Escherichia* (8.2%), *Pseudomonas* (7.3%), *Peptoniphilus* (4.9%) and *Serratia* (4.3%). The high‐level detection of *Escherichia* and other enteric species (31%, including *Enterobacter*, *Klebsiella*, *Stenotrophomonas* and *Serratia*) has also been reported in other studies [13, 17, 18, 22], which may be related to prior antibiotic therapy (19 patients). Similarly the levels of Gram positive opportunistic OTUs is high with *Staphylococcus*, *Streptococcus* and to a lesser extent *Enterococcus* at high levels, as has also been observed in DFU microbiome studies [13, 17, 18, 22].

Notably, the levels of Gram‐positive Anaerobic cocci (GPAC) sequences were prominent, with *Anerococcus*, *Finegoldia* and *Peptoniphilus* collectively representing 21.8% of all sequences combined (present at < 1% in 14 patients) as well as Gram negative anaerobes (*Prevotella*, 1.2%; and *Veilionella*, 4.4%). This may reflect the anaerobic nature of infective biofilms present in these wounds and has also been noted before [24, 25, 39, 40]. Additionally, sequences assigned to *Corynebacterium* (1.8%) were detected, highlighting a well‐known DFU and wound pathogen of clinical interest and increasing AMR significance [41, 42, 43].

**FIGURE 2:**
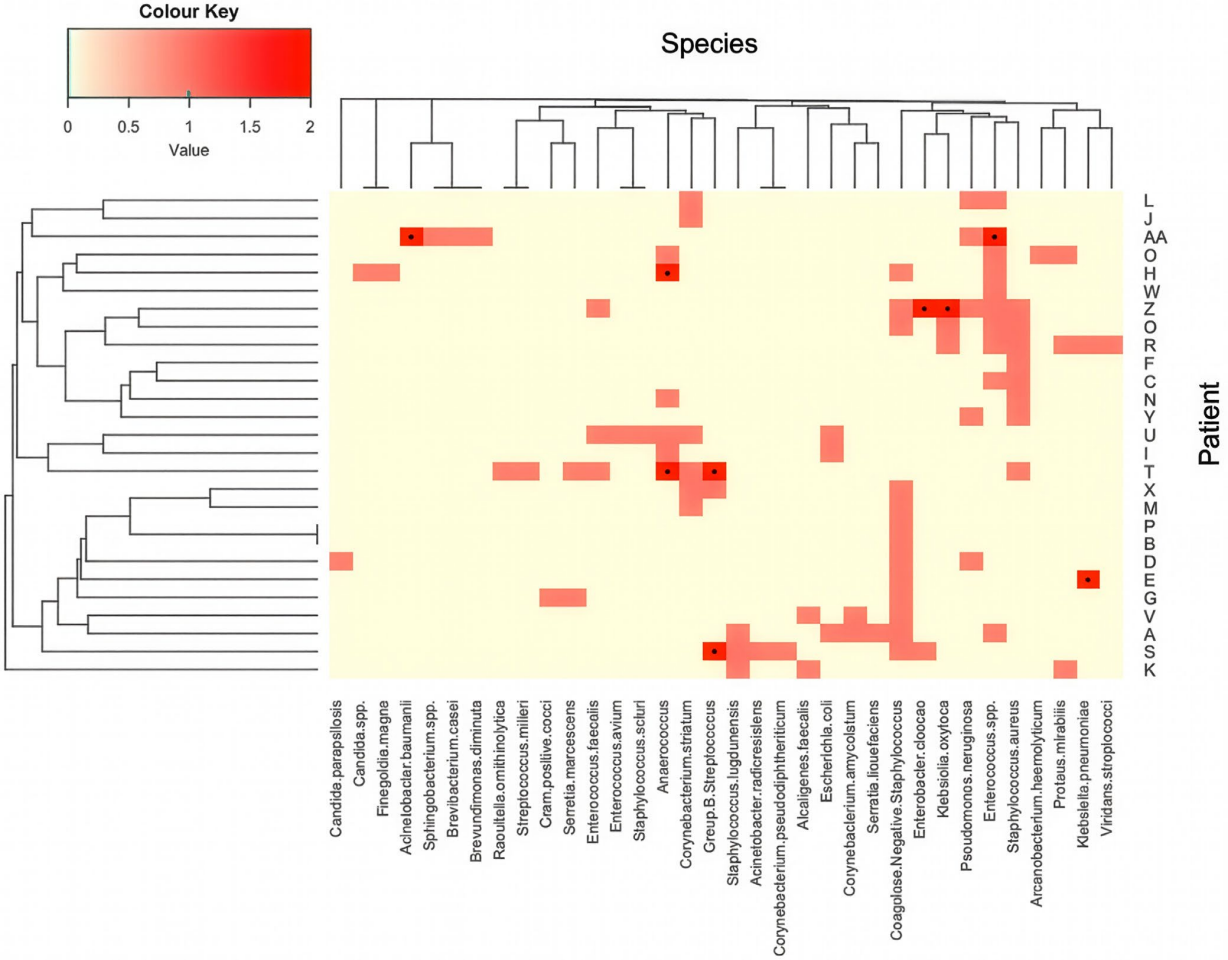
Summary of culture data: A total of 102 organisms were identified, with patient IDs represented by letter designations on the right. Strain species were identified using MALDI‐TOF and standard selective culture microbiology. Patients AA, E, H, S, T and Z were sampled on two visits with * indicating the presence of organisms in both samples; otherwise, isolates were only seen in the first visit. Clustering was performed using Jaccard Index and Hierarchical Clustering (Average Linkage) for both rows (patients) and columns (species) using heatmap.2 in R Studio.

When examined at patient level, only one of our patient samples (patient C) was dominated by one pathogen (patient C, *Pseudomonas*, Figure 1B, Figure S1A). However, several patient samples were dominated by Enteric sequences (A, E, G, I, U), *Staphylococcus* (Q, Z) and several by anaerobes (B, H). In contrast, the majority of the patient samples contain at least 3 spp. at abundance over 10%. Indeed, patient‐level calculations of diversity (Shannon: Figure S2A and Simpson: Figure S2B) indicate highly diverse populations > 0.5 (Simpson) in 15/21 (70%) of patient samples that correlate with the frequency data shown in (Figure S1A) and support the polymicrobial nature of the patient samples. PCA (Figure S1B) and hierarchical clustering (Figure 1B) analysis highlighted some potential clustering in the data (at least 4 groups, Figure S1B), but it is difficult to suggest reliable Community state Types without a larger data set. Notably, within our dataset, our two repeat patient samples (R and H, one month apart) have closely related communities (Figure 1B, Figure S1B). Finally, two of our patients (V, K) contained high levels (> 30%) of *Alcaligenes* sequences, which our culture data (below, Figure 2) would suggest are *Alcaligenes faecalis*, a species recently associated with accelerated healing in Diabetic wounds [44].

Overall, these data agree with several other studies of DFU where the DFU microbiome seems to reflect a complex polymicrobial community [24, 39, 40, 45]. While *Staphylococcus aureus* and CoNS species were among the most abundant, no two patients exhibited identical microbiomes, emphasising the complexity of DFU infections.

### 3.3 Culturome Data

In parallel to the swabs used for 16S amplicon sequencing, samples were collected as part of routine patient treatment for standard microbiological analysis, but with longer incubation times (48‐72 h) to culture anaerobic bacteria. This cultivation‐based approach (culturome) identified a total of 102 cultured organisms, which were identified using MALDI‐TOF, representing 34 different bacterial species, with an average of approx. 3.7 species per sample (Table S7) Patients AA, E, H, S, T and Z had isolates recovered on two separate visits. The most frequently isolated organisms by MALDI‐TOF were *Enterococcus* spp. (15%), CoNS (17% of all isolates) *Staphylococcus aureus* (8%), *Anaerococcus* (8%), *Corynebacterium striatum* (6%) and *Pseudomonas aeruginosa* (5%). These data largely correlated with the DNA sequencing data but allow us to add identify strains to species level—for example in the case of *Staphylococcus aureus* and CoNS where the sequence data only allows genus level resolution. The data also reveal several species of *Corynebacterium*, presence of *Alcaligenes faecalis* and an abundance of *Enterococcus faecalis* compared to the sequence data. The data also reveal a number of nosocomial enteric isolates such as *Escherichia*, *Klebsiella*, *Enterobacter*, *Serratia* and *Proteus* spp. (21% combined), were also recovered, consistent with sequence‐based observations.

In 25 of the 26 patients for which we received cultures (Figure 2), multiple species were cultured, indicating, like the DNA sequencing data, that Chronic DFU in this cohort is a polymicrobial infection. This picture broadly reflects our 16S sequence data, but also highlights the issues associated with culture as the only means to assess infection. For example, the extended anaerobic culture (5 days) allowed recovery of anaerobic Gram‐positive cocci (*Anaerococcus* and *Finegoldia*) in six of the patients, whereas no Gram‐negative anaerobes were recovered even though some present in the sequence data at low levels, for example *Prevotella* (1%. Table S1). Anaerobes are often slower growing in general and require isolation procedures that suppress fast‐growing facultative Gram‐positive bacterial growth and require extra nutrients (e.g., vitamin K, hemin) [46].

Culture‐based testing also identified two patients with *Candida* spp. (inc *C. parapsilosis* ) making up 2% of isolates. This agrees with recent work that identified *C. parapsilosis* as the most common fungal isolate from DFU in the United Kingdom, although the link to clinical outcome at this stage is unclear [47, 48].

### 3.4 Antibiotic Sensitivity Testing (AST) and Antibiotic Resistance Gene (ARG) Profiling

**FIGURE 3:**
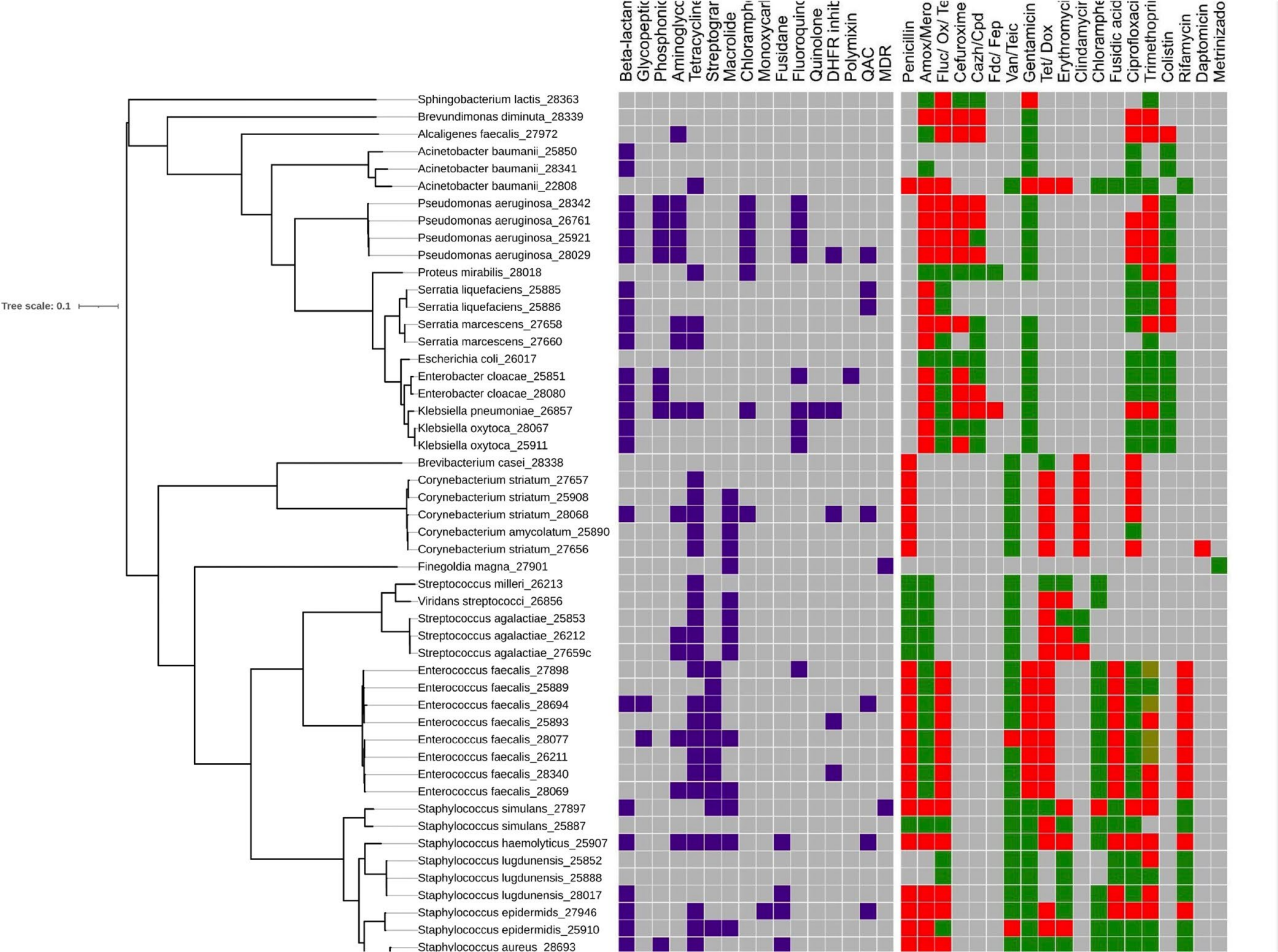
Summary of Genome sequencing, Antibiotic sensitivity and ARG presence in the Sheffield DFU clinical isolate collection. (Left) Phylogenetic tree of the Sheffield DFU clinical isolate collection (55 strains) across 18 patients. (Middle) Summary of the ARGs present in the collection, grouped by antibiotic class. The identification of an ARG is marked in purple and is presented in terms of the predicted antibiotic that the allele effects. (Right) Summary of the antimicrobial sensitivity testing (AST) of the collection. Heatmap shows sensitivity, resistance and intermediary as green, red and brown, respectively. Amox/Mero—Amoxicillin/Meropenem; Cazh/Cpd—Ceftazidime/Cefpodoxime; DHFR—Dihydrofolate Reductase; Fdc/Fep—Cefiderocol/Cefepime; Fluc/Ox/Tem—Flucloxacillin/Oxacillin/Temocillin; MDR—multi‐drug resistance; QAC—Quaternary ammonium compounds; Tet/Dox—Tetracycline/Doxycycline; Van/Teic—Vancomycin/Teicoplanin.

As part of the clinical collection of our isolates, routine Antibiotic Sensitivity Testing (AST) panels were used to test the clinical AMR profile of the isolates. As shown in Figure 3 (right) and Table S6 there were a wide range of AMR profiles in this culture collection, indicating that AMR strains are common in DFU. The most common AMR phenotypes included beta‐lactam resistance (AMC, AMX, PEN, FLUC, OXA and PTZ) across almost all genera except for the Streptococci. In addition, resistance to trimethoprim (30 isolates), ciprofloxacin (40) and cefuroxime (40) was also common (Table S6).

To enable us to investigate the AMR and genomic profile of our DFU isolate collection we attempted to resurrect clinical strains from frozen stocks and extracted the genomic DNA for NGS short‐read sequencing. The total revived in pure culture was 55, notably, only one of the 14 anaerobes was recovered from −80 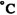 glycerol stock successfully. This reflects a possible need to go beyond the standard protocol of low volume (100 μL) bead‐based storage that is standard in the NHS. For example, our experience is that anaerobes need to be stored in rich media containing 15% glycerol with very dense culture and at least 1.5 mL in a 2 mL vial.

The completed genome sequences were phylogenetically compared based on whole genome nucleotide comparisons (Figure 3, left) showing this dataset contains representatives of 18 genera and 24 species. Overall, the data revealed a large number and range of ARGs across the collection (Figure 3, centre, Table S7). The most commonly occurring classes of ARG potentially encoded genes to resist tetracycline (tet(39, L, 38, K) efflux pumps and tet(W/O/M), ribosomal protection protein genes) and beta‐lactams (bla‐ many variants, see Table S8), observed in 56.3% and 45.5% of sequenced strains, respectively. The abundance of bla genes dominated with 58 bla ARGs detected among the 55 clinical isolates sequenced and included carbapenemase and cephalosporin resistance genes in *Pseudomonas* such as *blaOXA* [49] and *blaPDC* variants [50]. This aligns with the phenotypic AMR data; where the highest prevalence of resistance was against tetracycline/doxycycline (74.2%), penicillin (62.1%), flucloxacillin/oxacillin/temocillin (56.1%), trimethoprim (51.2%) and amoxicillin (50%) as well as ciprofloxacin and one strain with clinical vancomycin resistance ( *E. faecalis* 28,077 (*vanHAX*)).

Among the Gram‐negative enteric bacteria isolated, *Klebsiella pneumoniae* (28657) had multiple AMR phenotypes and ARGs, including the *blaCTX*‐M‐15 [51], which is associated with high level resistance to cephalosporins, including cefuroxime (CXM, Figure 3, Table S6). Notably, we also see phenotypic resistance to CXM in both *Klebsiella oxytoca* strains (25,911/28067), two of the *Serratia marcescens* (27,658/27660) strains and two of the *Enterobacter cloacae* (25,851/28080) isolates, probably due to Extended Spectrum Beta‐Lactamase of the AmpC [52], *blaACT* families [53]. Outside the enterics, all four of the *Pseudomonas aeruginosa* strains are multiply resistant to several antibiotics and contain a range of ARGs (*bla*, *aph*, *fos* genes), including several class D β‐lactamase genes of significant clinical significance (e.g., BLAOXA‐488. ‑486) [54, 55]. This data reflects the challenges posed by AMR Gram negatives in the United Kingdom, and it is evident here that these co‐occur in chronic DFU infections with AMR Gram positives such as *S. aureus* , CoNS and *Corynebacteria* in this cohort.

Finally, we also note ARGs for Quaternary Ammonium Compounds/disinfectant resistance (QAC) in nine strains across several genera including the well‐characterised *Staphylococcal qacA‐D* genes [56] and *qacE* in Corynebacteria [41], a concern potentially given their use as routine antiseptics in hospitals.

## 4 Conclusions

This study highlights the polymicrobial AMR profile of DFU infections in a cohort of chronic DFU patients in the United Kingdom. It also highlights that culture alone may significantly underestimate the presence of anaerobes in infected DFU, since we identified 90% of patients containing anaerobic genera OTU sequences, whilst only 12% contained anaerobes by culture. Furthermore, only one Gram‐positive anaerobe survived storage (2 m) before resurrection for sequencing, indicating the need to improve storage beyond standard protocols.

The high prevalence of AMR and ARGs observed in this DFU cohort is concerning, although it is likely influenced by the clinical background of these patients, many of whom had received multiple courses of antibiotics and had prolonged exposure to hospital environments, potentially creating selective pressure.

Finally, the data suggest the value of larger scale studies that could enable patient stratification by CSTs and maybe influence treatment planning. It is also important to note that this study did not account for the patients' inherent microbiomes, that is assess the correlation between organisms found in infected versus non‐infected wounds, as no samples from healthy sites were assessed. Future research could aim to validate findings across a broader patient cohort and investigate whether microbial profiles remain consistent over time in chronic DFU infection and ultimately lead to better‐targeted therapies and improved patient outcomes.

## Funding

This work was supported by Biotechnology and Biological Sciences Research Council, BB/T007222/1. NIHR Sheffield Biomedical Research Centre, NIHR203321.

## Ethics Statement

Swabbing of DFU wounds of heel ulcers and the isolation of the DFU wound microbiome were carried out under NHS ethics obtained by Dr. David Partridge (REC reference: 21/SC/0338 and IRAS project ID: 299735).

## Conflicts of Interest

The authors declare no conflicts of interest.

## Supporting information


**Figure S1:** (A) Overall relative abundance of taxa for each patient. The text labels have been placed for taxa that make up over 5% of the overall sample. The remaining genera were normalised so that the relative abundances sum to 100% within each patient sample. (B) Principal Components Analysis (Bray–Curtis, 70% confidence) indicating potential CSTs (1–4).
**Figure S2:** (A) The Shannon diversity index which reflects both species richness and evenness is shown for each patient swab sample. Higher values indicate a greater. Figure S2: (A) The Shannon diversity index which reflects both species richness and evenness is shown for each patient swab sample. Higher values indicate a greater microbial diversity within individual patients. Patient IDs are on the x‐axis of the plot. The diversity values were calculated in R Studio using the vegan package, specifically the diversity() function with ‘shannon’ index applied to normalised relative abundance data. (B) The Simpson diversity index which emphasises community evenness with a focus on the most dominant species is shown for each patient swab sample. The values are ranked out of 1, with a higher value representing a more evenly distributed microbial community. Patient IDs are represented on the x‐axis of the plot. The diversity values were calculated in R Studio using the vegan package, specifically the diversity() function with the ‘simpson’ index applied to normalised relative abundance data. The scale on the right‐hand side reflects the Simpson diversity values, with the legend indicating darker blue representing lower diversity and lighter blue represents higher diversity.

**Table S1:** Raw genus level count data for OTU from Patients in DFU cohort.

**Table S2:** Overall % abundance across all samples and % abundance over 1% across all samples.

**Table S3:** Summary data for culture. Each species, identified in each patient is indicated by number of isolates (by integer). Species ID was according to MALDI‐TOF Identification.

**Table S4:** % abundance of cultured isolates (> 1%): LEFT‐ grouped by genus and RIGHT by species.

**Table S5:** European Nucleotide archive accession numbers for microbome sequence data.

**Table S6:** Clinical Antibiotic Sensitivity testing data: S‐ sensitive, R‐ resistant, h‐High, I‐ intermediate. All according to EUCAST breakpoints.

**Table S7:** Antibiotic Resistance Gene(ARG) output from ResFinder, AMRFinderPlus with MLST where possible, resistance gene and predicted phenotype and actual phenotype from AST testing.

**Table S8:** Summary of ARGs in Beta‐lactam and Tetracycline groups and frequency of occurrence in sequenced genomes.

## Data Availability

The data that support the findings of this study are openly available in european nucleotide archive at https://www.ebi.ac.uk/ena/browser/home, reference number PRJEB98719.
