# Supplementary material for: A Survey of the Microbiome, Culturome and ARG Profile of a Cohort of Chronic Diabetic Foot Lesions
Source: APMIS. 2026 Apr 16;134(4):e70203. doi: 10.1111/apm.70203 (PMC13086565; doi:10.1111/apm.70203)
Supplement: Supplementary file 1 — Figure S1: (A) Overall relative abundance of taxa for each patient. The text labels have been placed for taxa that make up over 5% of the overall sample. The remaining genera were normalised so that the relative abundances sum to 100% within each patient sample. (B) Principal Components Analysis (Bray–Curtis, 70% confidence) indicating potential CSTs (1–4). Figure S2: (A) The Shannon diversity index which reflects both species richness and evenness is shown for each patient swab sample. Higher values indicate a greater. Figure S2: (A) The Shannon diversity index which reflects both species richness and evenness is shown for each patient swab sample. Higher values indicate a greater microbial diversity within individual patients. Patient IDs are on the x‐axis of the plot. The diversity values were calculated in R Studio using the vegan package, specifically the diversity() function with ‘shannon’ index applied to normalised relative abundance data. (B) The Simpson diversity index which emphasises community evenness with a focus on the most dominant species is shown for each patient swab sample. The values are ranked out of 1, with a higher value representing a more evenly distributed microbial community. Patient IDs are represented on the x‐axis of the plot. The diversity values were calculated in R Studio using the vegan package, specifically the diversity() function with the ‘simpson’ index applied to normalised relative abundance data. The scale on the right‐hand side reflects the Simpson diversity values, with the legend indicating darker blue representing lower diversity and lighter blue represents higher diversity. [file APM-134-0-s005.pdf]

A.

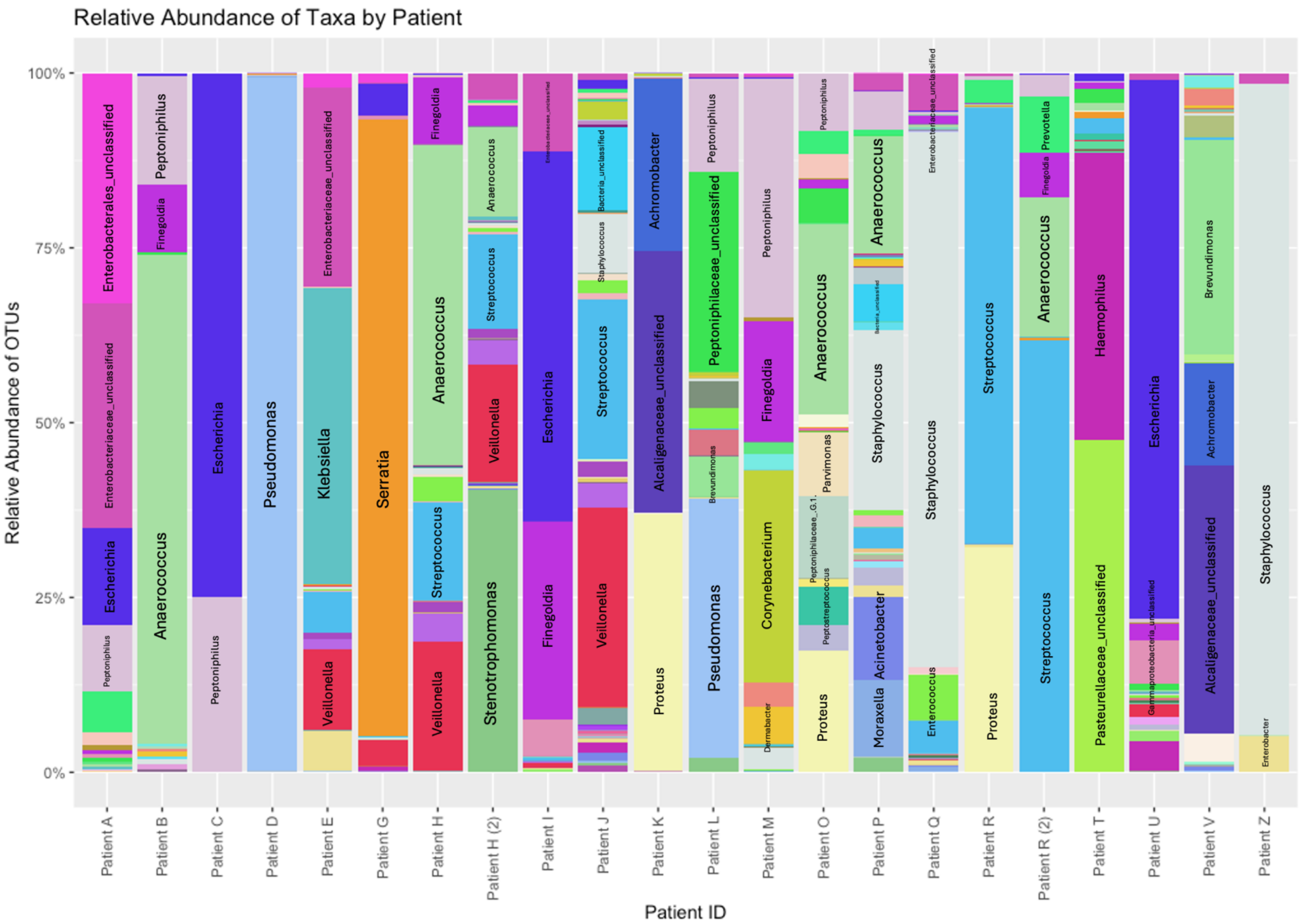

B.

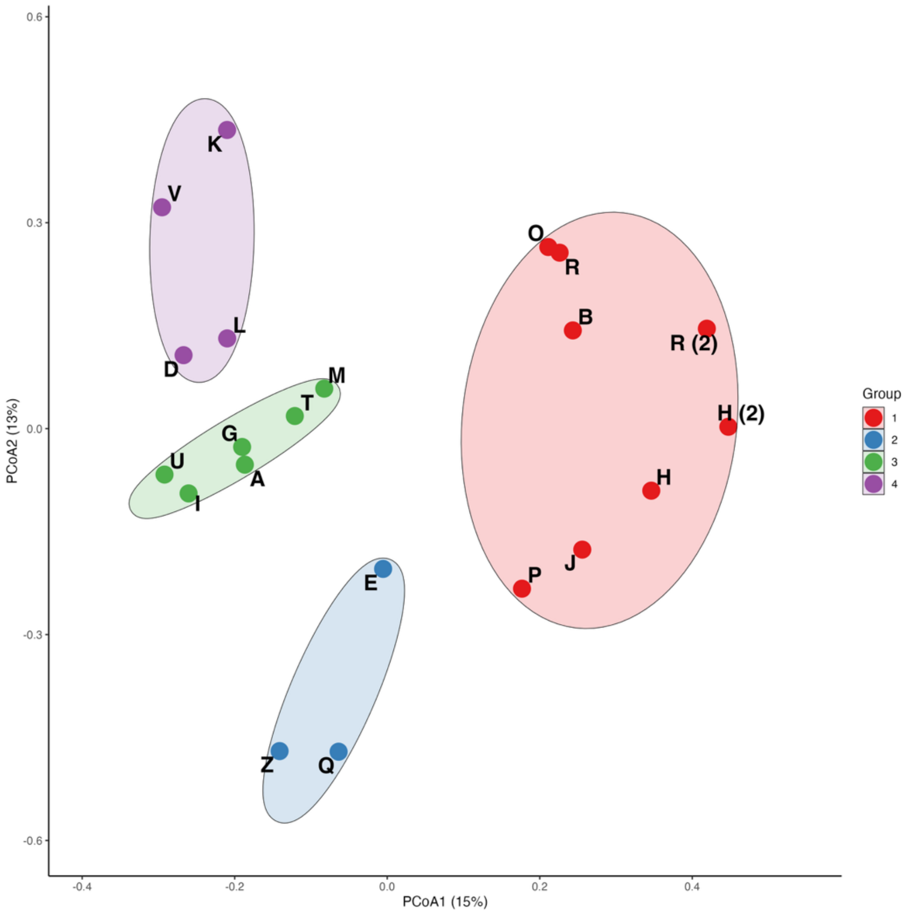

Figure S1. A) Overall relative abundance of taxa for each patient. The text labels have been placed for taxa that make up over 5% of the overall sample. The remaining genera were normalised so that the relative abundances sum to 100% within each patient sample. **B)** Principal Components Analysis (Bray-Curtis, 70% confidence) indicating potential CSTs (1-4).

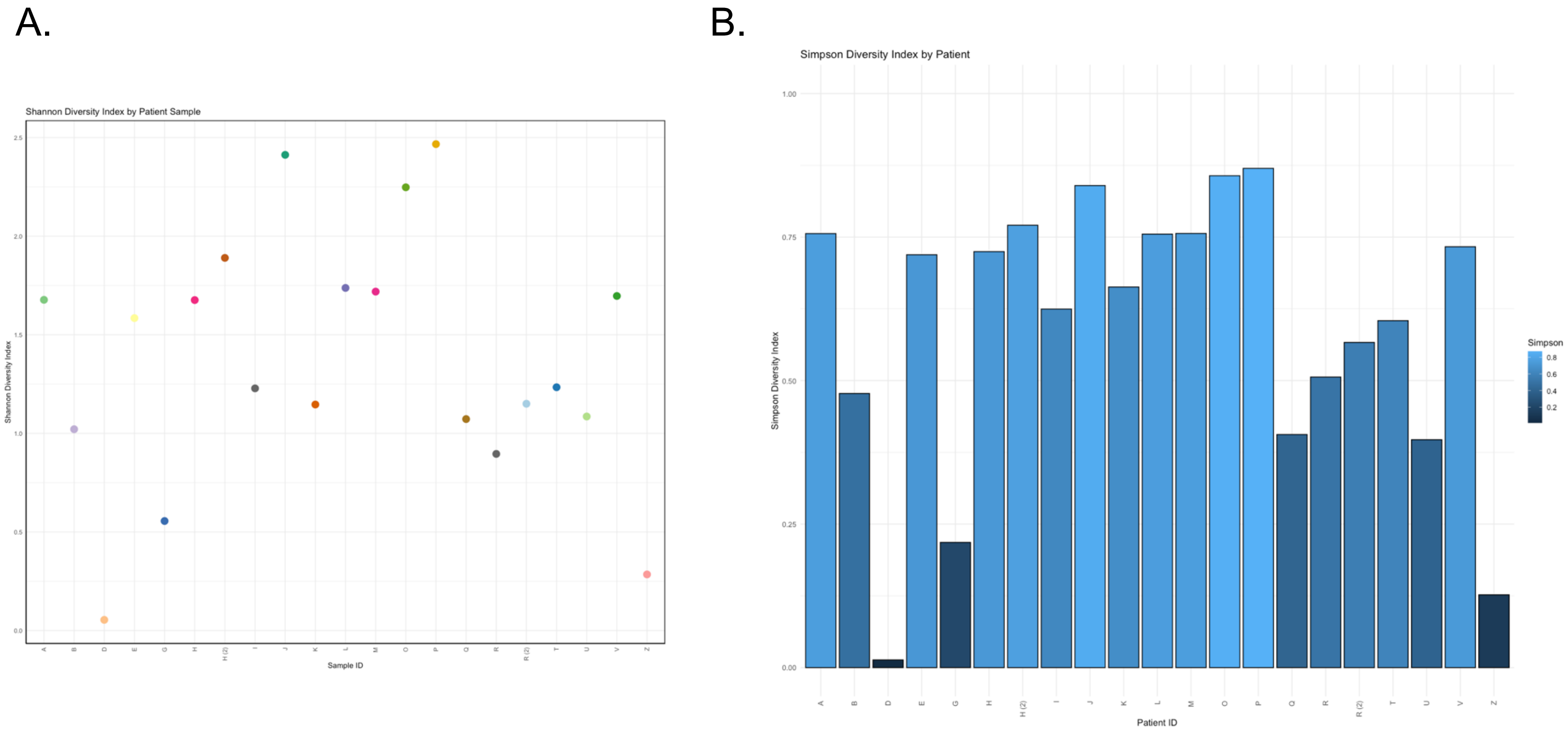

Figure S2. A) The Shannon diversity index which reflects both species richness and evenness is shown for each patient swab sample. Higher values indicate a greater microbial diversity within individual patients. Patient IDs are on the x-axis of the plot. The diversity values were calculated in R Studio using the vegan package, specifically the diversity() function with “shannon” index applied to normalised relative abundance data. **B)** The Simpson diversity index which emphasises community evenness with a focus on the most dominant species is shown for each patient swab sample. The values are ranked out of 1, with a higher value representing a more evenly distributed microbial community. Patient IDs are represented on the x-axis of the plot. The diversity values were calculated in R Studio using the vegan package, specifically the diversity() function with the “simpson” index applied to normalised relative abundance data. The scale on the right-hand side reflects the Simpson diversity values, with the legend indicating darker blue representing lower diversity and lighter blue represents higher diversity.
